# There is no evidence that carbon dioxide-enriched oxygen before apnea affects the time to arterial desaturation, but it might improve cerebral oxygenation in anesthetized obese patients: a single-blinded randomized crossover trial

**DOI:** 10.1186/s12871-023-01982-9

**Published:** 2023-02-06

**Authors:** Marc T. Schmidt, Marc Studer, Andres Kunz, Sandro Studer, John M. Bonvini, Marco Bueter, Lucas Kook, Sarah R. Haile, Andreas Pregernig, Beatrice Beck-Schimmer, Martin Schläpfer

**Affiliations:** 1 Institute of Anesthesiology, University Hospital Zurich, Zurich, Switzerland; 2 Swiss Air Force, Bern, Switzerland; 3 Clinical Trials Center, University Hospital Zurich, Zurich, Switzerland; 4 Department of Surgery, University Hospital Zurich, Zurich, Switzerland; 5 Epidemiology, Biostatistics and Prevention Institute, University Zurich, Zurich, Switzerland; 6 Department of Anesthesiology, University of Illinois at Chicago, Chicago, USA; 7 Institute of Physiology, Zurich Center for Integrative Human Physiology, University Zurich Irchel, Zurich, Switzerland

**Keywords:** Hypoxia, apnea tolerance, tissue oxygenation, cerebral oxygenation

## Abstract

**Purpose:**

Carbon dioxide (CO_2_) increases cerebral perfusion. The effect of CO_2_ on apnea tolerance, such as after anesthesia induction, is unknown. This study aimed to assess if cerebral apnea tolerance can be improved in obese patients under general anesthesia when comparing O_2_/Air (95%O_2_) to O_2_/CO_2_ (95%O_2_/5%CO_2_).

**Methods:**

In this single-center, single-blinded, randomized crossover trial, 30 patients 18–65 years, with body mass index > 35 kg/m^2^, requiring general anesthesia for bariatric surgery, underwent two apneas that were preceded by ventilation with either O_2_/Air or O_2_/CO_2_ in random order. After anesthesia induction, intubation, and ventilation with O_2_/Air or O_2_/CO_2_ for 10 min, apnea was performed until the cerebral tissue oxygenation index (TOI) dropped by a relative 20% from baseline (primary endpoint) or oxygen saturation (SpO_2_) reached 80% (safety abortion criterion). The intervention was then repeated with the second substance.

**Results:**

The safety criterion was reached in all patients before cerebral TOI decreased by 20%. The time until SpO_2_ dropped to 80% was similar in the two groups (+ 6 s with O_2_/CO_2_, 95%CI -7 to 19 s, *p* = 0.37). Cerebral TOI and PaO_2_ were higher after O_2_/CO_2_ (+ 1.5%; 95%CI: from 0.3 to 2.6; *p* = 0.02 and + 0.6 kPa; 95%CI: 0.1 to 1.1; *p* = 0.02).

**Conclusion:**

O_2_/CO_2_ improves cerebral TOI and PaO_2_ in anesthetized bariatric patients. Better apnea tolerance could not be confirmed.

**Supplementary Information:**

The online version contains supplementary material available at 10.1186/s12871-023-01982-9.

## Introduction

Before general anesthesia induction, patients are administered 100% oxygen (O_2_) to prevent hypoxia and brain damage should ventilation fail. In the blood, oxygen is transported predominantly (≥ 98%) bound to hemoglobin within red blood cells [1]. Increased carbon dioxide partial pressure (PCO_2_), temperature, and decreased pH, lower O_2_ hemoglobin affinity [2]. In the systemic circulation, high CO_2_ and low pH promote vasodilatation. Metabolically active tissue is; therefore, better perfused, and oxygen release is facilitated. Hypercarbia and acidemia are essential components of cerebral autoregulation [3]. Hypoxia stimulates vasodilation. This effect is amplified by the more potent vasodilator CO_2_; thus, cerebral blood flow increases more than with hypoxia alone [4]. Cerebral oxygenation and task performance of rhesus monkeys subjected to hypoxia improved under the influence of an admixture of CO_2_ [5], which may be a result of the above-mentioned mechanisms.

To date, functional effects of CO_2_ have been primarily described for hypobaric hypoxic conditions, such as in high altitude, aviation, and aerospace medicine [6–8].

Improved task performance with an increased CO_2_ partial pressure has also been described under normobaric hypoxia [8]. A higher CO_2_ partial pressure increases cerebral blood flow [9], which results in a higher TOI [10]. Whether higher CO_2_ concentrations improve cerebral oxygenation in anesthetized humans and protect the brain from impending hypoxia, respectively, prolonging the time for a defined TOI drop is unclear. Obese patients have a decreased oxygen reserve due to a low functional residual lung capacity (FRC), are therefore at risk for hypoxia, and might benefit from CO_2_ application. Low TOI may be associated with delirium [11], but no TOI and time thresholds have been established yet. Clinical studies on apnea tolerance during anesthesia induction are almost nonexistent [12], let alone in obese patients. One study reported that the incidence or a relevant drop of peripheral oxygen saturation (SpO_2_) to < 80% could be observed in 8% of obese patients undergoing bariatric surgery [13]. This is all the more relevant as obesity is a risk factor for difficult mask ventilation [14].

This current study aimed to assess if cerebral apnea tolerance can be improved with the use of O_2_/CO_2_ compared to O_2_/Air in obese patients under general anesthesia. We hypothesized that ventilation with O_2_/CO_2_ would extend apnea time until cerebral tissue oxygenation index (TOI = regional oxygen saturation, SrO_2_) decreased by a relative 20% from baseline.

## Materials and methods

### Trial design

This is a single-center, single-blinded, proof-of-concept randomized crossover trial.

### Participants, eligibility criteria, setting

This study was approved by the local ethics committee (Kantonale Ethikkommission Zurich, Zurich, Switzerland, number 2017 – 01,790) as well as by the national authorization and supervisory authority for drugs and medical products (Swissmedic Bern, Switzerland, notification number 2017DR2183).

Inclusion criteria were defined as patients between 18 and 65 years old being scheduled for primary bariatric surgery with a BMI > 35 kg/m^2^ and signed informed consent to participate in this study. Exclusion criteria were severe end-organ damage (chronic obstructive pulmonary disease GOLD III and IV or other chronic respiratory diseases; known hepatic insufficiency or liver enzymes > 50% over the upper reference value of the University Hospital Zurich; renal creatinine clearance < 30 ml/min; diagnosed pulmonary hypertension (mean pulmonary arterial pressure ≥ 25 mmHg); severe cardiovascular disease (New York Heart Association, NYHA classification III and IV); history of cerebrovascular disease; drug- or alcohol abuse; and pregnancy). In patients at risk for cardiovascular disease (age > 50 years, history of atherosclerosis, or any related diseases), significant vascular stenosis (> 50%) of the carotid arteries was excluded by duplex examination.

All participants of the study provided written and informed consent to participate in this trial. It was registered before patient enrollment on www.clinicaltrials.gov (NCT 03,338,907; date of registration: 09/11/2017) and is reported according to the consolidated standards of reporting trials (CONSORT) checklist [15]. The study was monitored by the clinical trials center of the University Hospital Zurich, Zurich, Switzerland. The trial was performed at the University Hospital Zurich between January and November 2018.

### Randomization and blinding

Patients were randomized on the day of their scheduled surgery using a web-based randomizer (www.randomizer.at) which allowed allocation concealment. Patients were randomized (1:1) using simple randomization to the intervention sequence O_2_/Air—O_2_/CO_2_ or to O_2_/CO_2_—O_2_/Air. The allocation sequence was generated electronically. The parameters to generate the allocation sequence have been determined by the biostatistician. The participants were enrolled by one of the investigators and were assigned to the intervention sequence by the web-based randomizer.

### Participant preparation for anesthesia and anesthesia induction

Upon arrival in the operating theater, patients were monitored by the study team with peripheral oxygen saturation (SpO_2_) monitoring, electrocardiogram (ECG), venous and arterial catheters, bispectral index (BIS), and near-infrared spectroscopy (NIRS, Sensmart X-100, Nonin Medical, Plymouth, MN, USA) for tissue oxygen index (TOI) measurement. The NIRS and BIS sensors were placed on the patient’s left and right foreheads, respectively.

Patients were preoxygenated with 100% O_2_ until an expiratory fraction of oxygen of ≥ 90% was reached, after which rapid sequence induction according to departmental guidelines was performed with intravenous fentanyl 2–3 µg/kg, propofol 2 mg/kg, and rocuronium 0.9 mg/kg. The airway was secured by an endotracheal tube placed with a C-MAC video laryngoscope (Storz, Tuttlingen, Germany). General anesthesia was then maintained with propofol 4–10 mg/kg/h, and BIS monitoring was used to ensure a constant depth of anesthesia throughout the study intervention (BIS target range 30–50).

### Study intervention, definition of the time-points T0-T4

#### T0

**Fig. 1:**
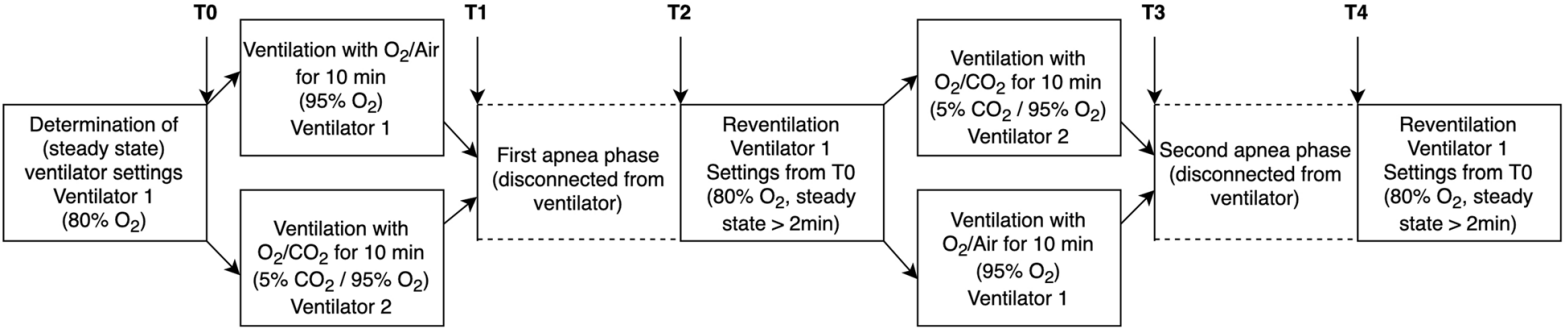
Study intervention. This graphic displays the patient’s study intervention

An overview of the study intervention is given in Fig. 1. After induction, patients were ventilated (Dräger Primus Infinity Empowered, Dräger, Lübeck, Germany) in a volume-controlled mode with an inspiratory oxygen fraction (FiO_2_) of 0.8. Tidal volume was set at 6–8 ml/kg ideal body weight. At peak pressure above 35 cmH_2_O, tidal volume was reduced so that this value was not exceeded. Peak inspiratory pressure was 15–35 cmH_2_O, and the respiratory rate was 10–18 breaths per minute to obtain normocapnic end-tidal CO_2_ (EtCO_2_) values of 4.5–5.5 kPa for ≥ 2 min. This steady state was defined as time-point 0 (T0), and baseline values and ventilator settings were recorded. The ventilator setting established at T0 was maintained for each patient throughout the experiment.

#### T1 and T2

Patients were then first connected either to O_2_/Air (administered by a Dräger Primus Infinity Empowered ventilator, connected to the hospital’s O_2_ and compressed air wall supply) or to O_2_/CO_2_ (administered by a Dräger Evita Infinity V500 ventilator, connected to an O_2_/CO_2_ gas bottle, Linde Group, Dublin, Ireland).

Patients were ventilated for ≥ 10 min with O_2_/Air or O_2_/CO_2_; the fraction of expired O_2_ (FeO_2_) had to reach ≥ 80% and be stable for more than one minute. To initiate apnea, the endotracheal tube was disconnected from the ventilator. T1 was the initiation of the first apnea phase. Ventilation was resumed when TOI had dropped 20% from baseline (primary endpoint). The 20% drop was defined as a relative drop from the baseline and not an absolute 20% drop. This value was chosen according to institutional standards and expert recommendations for carotid endarterectomy [16]. For safety reasons, the apnea phase was terminated early if a SpO_2_ of 80% was reached (safety endpoint).

T2 was defined as the time point at the end of the first apnea phase (when the primary or the safety endpoint was reached). Ventilation was performed with the ventilator settings used at T0 to allow the patient to return to its steady state for ≥ 2 min. Then the crossover ventilation was initiated for 10 min.

#### T3 and T4

T3 corresponds to the beginning of the second apnea phase, and T4 to the end of the second apnea phase.

### End of study intervention and follow-up

After T4, upon reaching a steady state, the study intervention was completed, and bariatric surgery was started. To exclude study-related adverse (AE) or serious adverse events (SAE), each patient was followed-up after 24 h. In the case of an AE or SAE, the patient was followed-up until the AE or SAE was resolved.

### Outcomes

The primary outcome of this study was the length of the apnea phase until TOI decreased by 20% from baseline (assessed by NIRS). Differences between the start and stop of apnea phases were measured for PaO2, PaCO2, SpO2, and TOI (also if the primary endpoint could not be reached), BIS values, heart rate, and mean arterial pressure (MAP) were defined as secondary endpoints.

At all time points (T0 to T4), blood gas samples were obtained and measured using the ABL835 Flex blood gas analyzer from Radiometer Medical (Copenhagen, Denmark).

### Sample size calculation

For the power calculation, data variability has been estimated using data from Eichhorn et al. [17] on the primary outcome (length of the apnea phase in s until TOI decreased by 20% from baseline). Assuming a within-subject correlation of *ρ* = 0.3, a standard deviation of 45.04 s was calculated [18]. We determined that a mean difference of 30 s in the primary outcome would be clinically relevant. Using the formula published by Senn [19], a total of 28 patients would be needed to show an average difference of 30 s between the two treatments. Assuming a dropout rate of 5%, the target sample size of 30 was determined.

### Blinding

Patients were blinded to the intervention. The biostatistician was blinded for the analysis. The investigators were not blinded.

### Statistical analyses

Continuous data were summarized as median and interquartile range (IQR), and categorical data were summarized as numbers (n) and proportions of the total (%). As specified in the study protocol, linear mixed-effects models were used to estimate the average effect of O_2_/CO_2_ adjusted for potential period effects while including the patient ID as a random effect. Other models were additionally adjusted for baseline measurements or the presence of obstructive sleep apnea. As baseline correction did not impact the study results, all data are presented without baseline correction in the final analyses.

Linear mixed models with and without adjustment for additional covariables, model diagnostics, as well as visualization methods adhere to the recommendations published by Senn [19]. Pearson correlation was used to quantify the linear association between study outcomes at T2 and T4. As presumed in the trial protocol, missing data did not exceed 5% in any of the primary or secondary outcomes. While no imputation methods were considered, the models included all patients, even if missing values were present in one of the two intervention periods. All analyses were performed in the R programming language (R Core Team, 2017) (R version 3.5.2 (2018–12-20)). Linear mixed-effects models were fit using the lme4 package [20], with p-values computed using the lmerTest package [21].

## Results

**Fig. 2:**
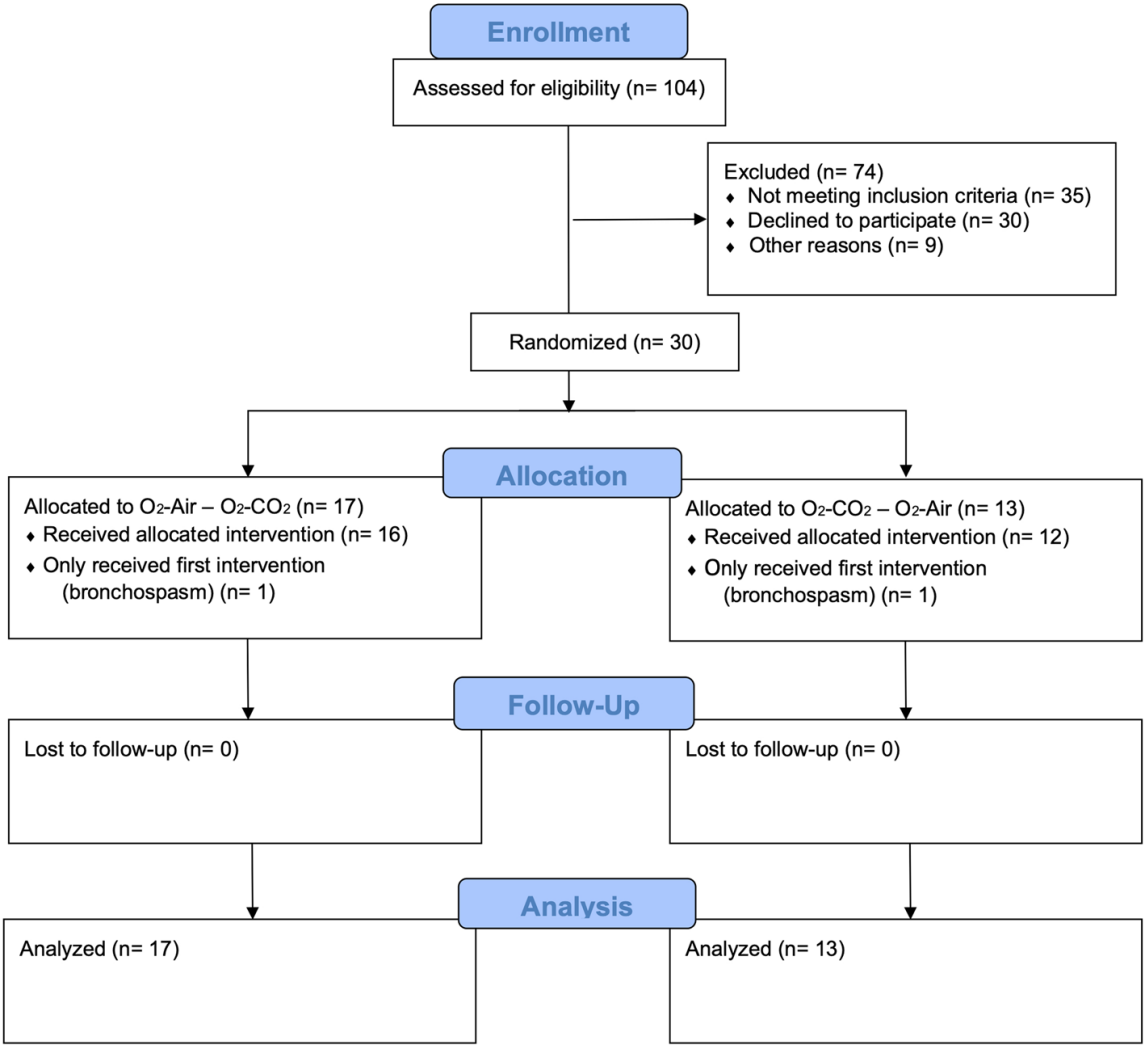
CONSORT flowchart diagram. The adapted CONSORT flowchart diagram shows the patient flow in this cross-over trial

Between January 24 and October 26, 2018, 104 patients scheduled for bariatric surgery at the University Hospital Zurich were screened for eligibility, of which 30 were enrolled in the study. Seventeen patients were randomized to receive O_2_/Air followed by O_2_/CO_2_, and 13 patients to receive O_2_/CO_2_ followed by O_2_/Air. The patient flow is depicted in Fig. 2.

### Data collection and presentation

Two patients, one in the O_2_/Air—O_2_/CO_2_ group and one in the O_2_/CO_2_—O_2_/Air group, could not undergo the second intervention due to bronchospasm during the first re-ventilation phase. Two patients started to breathe spontaneously during the apnea phase, and in one patient, a blood sample was drawn not at the end of the apnea phase but when re-ventilation had already started.

### Patient characteristics and baseline parameters

Patient characteristics, including age, sex, BMI, as well as comorbidities (arterial hypertension, diabetes, NYHA class,) and laboratory parameters, were balanced between the two randomization sequences, except for the incidence of sleep apnea, which was higher in the O_2_/Air—O_2_/CO_2_ than in the O_2_/CO_2_—O_2_/Air sequence. All patient characteristics are presented in Table 1.

**Table 1:** Patient characteristics

		O_2_/Air – O_2_/CO_2_	O_2_/CO_2_ – O_2_/Air
n		17	13
Age (years)		41 [31, 51]	43 [34, 52]
Sex	female	11 (65)	10 (77)
BMI (kg/m^2^)		45.0 [40.6, 51.4]	42.6 [40.8, 47.1]
Arterial hypertension (n)		9 (53)	6 (46)
Diabetes (n)		2 (12)	2 (15)
Sleep apnea (n)		9 (53)	2 (15)
NYHA class (n)	0	14 (82)	11 (84)
	1	0 (0)	1 (8)
	2	3 (18)	1 (8)
Hemoglobin (g/l)		143 [132, 150]	138 [125, 145]
Creatinine (μmol/l)		70 [59, 87]	68 [64, 84]

Data reported as absolute number (percentage) and median [Q1, Q3] for the two intervention sequences O_2_/Air—O_2_/CO_2_ and O_2_/CO_2_—O_2_/Air

At baseline (T0), TOI, BIS values, MAP, HR, SpO_2_, EtCO_2_, respiratory rate, airway pressures, PaCO_2_, and PaO_2_ were comparable between the two randomization sequences. At the same time, the O_2_/Air-first group had a higher FiO_2_ and minute ventilation than the O_2_/CO_2_—O_2_/Air group. Detailed information regarding the neurological activity, vital parameters, and ventilator settings at T0 are provided in Table 2.

**Table 2:** Baseline values and settings at T0

	O_2_/Air – O_2_/CO_2_	O_2_/CO_2_ – O_2_/Air
n	17	13
TOI (%)	82 [78, 84]	81 [79, 84]
BIS	38 [29, 42]	35 [27, 40]
MAP (mmHg)	69 [63, 78]	71 [67, 74]
Heart Rate (min^−1^)	71 [66, 84]	80 [74, 93]
SpO_2_ (%)	98 [97, 98]	98 [96, 98]
FiO_2_ (%)	84 [80, 92]	80 [77, 80]
ETCO_2_ (%)	4.8 [4.6, 5.0]	4.9 [4.7, 5.1]
Respiratory rate (min^−1^)	14 [13, 16]	13 [12, 14]
MV (l/min)	7.1 [6.4, 8.0]	6.0 [5.9, 6.4]
PEEP (cmH_2_O)	8 [7, 8]	8 [7, 8]
PIP (cmH_2_O)	23 [21, 27]	22 [20, 23]
PaCO_2_ (kPa)	5.15 [5.06, 5.72]	5.49 [5.04, 5.65]
PaO_2_ (kPa)	24.9 [18.2, 36.7]	25.7 [24.1, 27.6]

Data reported as median [Q1, Q3]. Data described in Table 2 were recorded after reaching a steady state at time-point T0 when ventilating patients with a fraction of inspired oxygen (FiO_2_) of 80% before starting the wash-in of the first study gas

*Abbreviations:* *n* numbers, *TOI* cerebral tissue oxygenation index (= regional cerebral saturation of oxygen, SrO_2_), *BIS* bispectral index, *MAP* mean arterial pressure, *SpO*_*2*_ peripheral oxygen saturation, *FiO*_*2*_ fraction of inspired oxygen, *ETCO*_*2*_ end-tidal carbon dioxide, *MV* minute volume, *PEEP* positive end-expiratory pressure, *PIP* peak inspiratory pressure, *PaCO*_*2*_* and PaO*_*2*_ partial pressure of arterial CO_2_ and O_2_

### Primary and secondary outcomes

**Fig. 3:**
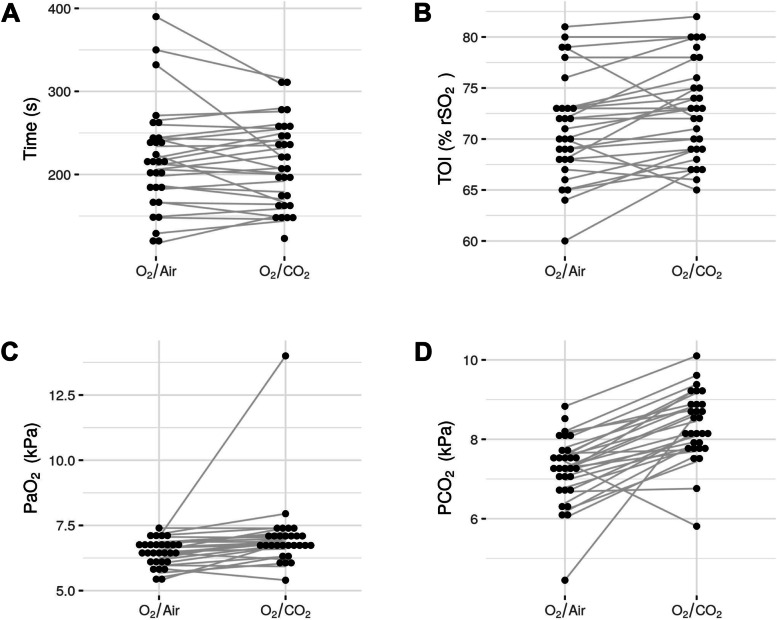
Values at the end of the apnea. The time until the peripheral oxygen saturation (SpO_2_) reached 80% (**A**), cerebral tissue oxygenation index (TOI) (**B**), arterial oxygen pressure (PaO_2_) (**C**), and arterial carbon dioxide pressure (PaCO_2_) (**D**) at the end of the apnea phase are displayed. The time until SpO2 reached 80% was similar after the two treatments, while TOI (*p* = 0.02), PaO_2_ (*p* = 0.02), and PaCO_2_ (*p* < 0.001) increased under O_2_/CO_2_ administration

The primary outcome, time until TOI dropped by 20%, was not a feasible target. The safety criterion for early termination of the apnea—SpO_2_ dropping to 80%—was reached first in all patients (*n* = 30). The primary outcome, time until TOI dropped by 20%, could thus not be measured. The time until SpO_2_ dropped to 80% was similar after ventilation with O_2_/CO_2_ vs. O_2_/Air (+ 6 s; 95%CI: from -7 to 19; *p* = 0.37; Fig. 3A).

**Fig. 4:**
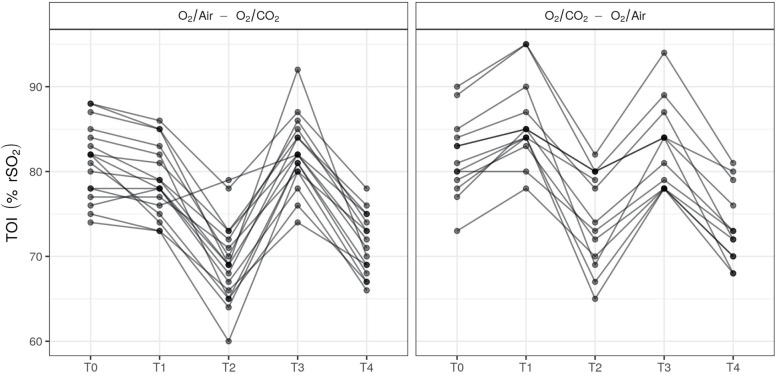
TOI-values at the different time points. The graph illustrates the brain tissue oxygenation (TOI) values in the sequence O_2_/Air—O_2_/CO_2_ (A, *n* = 17) and the sequence O_2_/CO_2_—O_2_/Air (B, *n* = 13)

At the end of the apnea phase (T2 and T4, respectively), cerebral TOI was significantly higher after O_2_/CO_2_ compared to O_2_/Air, with a mean difference of + 1.5% (95%CI: from 0.3 to 2.6; *p* = 0.02; Figs. 3B and 4). Similarly, O_2_/CO_2_ was also associated with a higher PaO_2_ (at T2 and T4, respectively, mean difference + 0.6 kPa; 95%CI: from 0.1 to 1.1; *p* = 0.02; Fig. 3C) and higher PaCO_2_ at the end of the apnea phase (mean difference + 1.06 kPa; 95%CI: from 0.76 to 1.36; *p* < 0.001; Fig. 3D).

BIS, MAP, and HR were comparable after ventilation with O_2_/CO_2_ (vs. O_2_/Air) (BIS: + 1%; 95%CI: from -3 to 5; *p* = 0.7; MAP: -3 mmHg MAP; 95%CI: from -9 to 3; *p* = 0.3; HR: + 1/min; 95%CI: from -1 to 4; *p* = 0.3). Details about the treatment effects on all primary and secondary outcomes are reported in Table 3.

**Table 3:** O_2_/CO_2_ treatment effects on primary and secondary outcomes

Outcome	Treatment effect	Confidence interval	*p*-value
BIS	0.92	from -3.23 to 5.07	0.67
HR	1.42	from -0.93 to 3.73	0.25
MAP	-3.17	from -9.24 to 2.90	0.31
PaO_2_	0.60	from 0.12 to 1.09	0.02
PaCO_2_	1.06	from 0.76 to 1.36	< 0.001
SpO_2_	-0.01	from -0.49 to 0.48	0.98
Time	-5.94	from -18.73 to 6.84	0.37
TOI	1.46	from 0.33 to 2.59	0.02

The table shows the treatment effects of O_2_/CO_2_ on primary, secondary, and exploratory outcomes obtained by linear mixed-effects models without baseline adjustments. Fixed effects are reported with 95%-confidence interval and *p*-value

Abbreviations: *BIS* bispectral index, *HR* heart rate (per minute), *MAP* mean arterial pressure (in mmHg), *PaO2* arterial partial pressure of oxygen (in kPa), *PaCO2* arterial partial pressure of carbon dioxide, *SpO2* peripheral oxygen saturation (in %), *time* time until the safety outcome was reached, *TOI* tissue oxygenation index

Additionally, the lowest TOI value for both interventions was defined in each subject, and the time to reach it after apnea initiation was determined. The time to the lowest TOI was similar for O_2_/CO_2_ vs. O_2_/Air (mean: + 9 s; 95%CI: from -2 to 20; *p* = 0.1). Details are available in the supporting information S1.

### Adverse events

Two patients had bronchospasm after the first apnea phase and were not subjected to a second apnea phase. Two individuals complained of postoperative thoracic pain and one of epigastric pain. Cardiac involvement could be excluded by electrocardiogram and troponin T measurement in all patients.

## Discussion

This study assessed the effect of O_2_/CO_2_ to improve cerebral oxygenation and apnea tolerance in obese patients under general anesthesia. While the primary endpoint could not be reached due to safety reasons, our results indicate improved cerebral TOI and a higher PaO_2_ after ventilation with O_2_/CO_2_. However, the time until SpO_2_ dropped to the safety threshold of 80%, as well as BIS, MAP, and HR values, were similar after O_2_/Air and O_2_/CO_2_ application. Ventilation with O_2_/CO_2_ did not affect the time to the lowest TOI measured compared to O_2_/Air.

The benefits of CO_2_ on brain oxygenation and functional outcomes, such as task performance, have mainly been the subject of high-altitude or aviation research. These outcome changes might result from a right shift of the oxygen dissociation curve [22], a reduction in pulmonary shunting [23], or an increase in cardiac output [24] in response to a higher CO_2_ partial pressure. Most important, however, is probably cerebral tissue oxygenation, which is affected by CO_2_-dependent vasodilation [25].

Obese patients represent a population particularly vulnerable to hypoxia as they have a reduced oxygen reserve due to a lower FRC. Indeed, even after pre-oxygenation, obese patients present with a clinically relevant desaturation to 90% in less than three minutes, which is much faster compared to non-obese patients [26, 27]. As these patients have such a low tolerance for apnea, even a slight advantage provided by CO_2_ could benefit and help to prevent neurological sequelae if scenarios such as “cannot ventilate, cannot intubate” occur.

A strength of our study was the crossover study design, which increases the precision of the treatment effect measurement and minimizes confounding, as each patient acts as their control.

Our study, however, has some limitations. The primary outcome, the length of the apnea phase until TOI decreased by a relative 20% from baseline, could not be reached due to safety reasons. Changing a primary endpoint after the start of a clinical trial is only justifiable if the reasons for doing so are based on new information unrelated to the study conducted, for example, from another independent study [28]. Since this was not the case, the primary endpoint could not be changed. Nevertheless, all data from clinical trials should be published. Our data could lay the ground for studies with a more accessible primary endpoint. Furthermore, the crossover design of this proof-of-concept study allowed for a smaller sample size in a single-center trial. A further limitation of this study was that only patients and the biostatistician were blinded, while the investigators were not blinded.

For this study, we defined our primary endpoint as a relative 20% cerebral TOI reduction from baseline. A larger decrease has a negative predictive value in carotid surgery [16]. Experimental studies in piglets suggest that immediate damage is unavoidable if TOI drops below 30–45%. Above this value, which has not been established nor confirmed in humans, the onset of injury appears to be time-dependent [29]. The benefit of CO_2_ could, therefore, be more significant at different thresholds or under more severe conditions of hypoxia.

The safety abortion criterion was chosen so that a clinically relevant level of hypoxia was reached while ensuring the patient’s safety, based on previous data. While data on anesthetized patients are scarce, hypoxia studies in awake probands exposed to SaO_2_ levels of < 80% for 15 min [29] and hypoxia to SaO_2_ levels of 50–70% for 10 to 30 min were well tolerated and not associated with adverse outcomes in healthy adults [30]. Data from healthy subjects cannot be extrapolated to multimorbid patients, such as morbidly obese patients. A recent systematic review in sedated ICU patients suggests that low TOI is associated with a higher incidence of delirium in critically ill patients [11]. The duration of low TOI is likely to be important. Because the duration of measurement has not been reported in many studies and TOI values are variable [11]. a safety threshold has not yet been established. A relative decrease in TOI of 20% from baseline, established in patients diagnosed with cerebrovascular disease and undergoing carotid endarterectomy, was proposed as the primary endpoint of this study [16]. Because the authors decided to resume ventilation immediately after the primary endpoint or safety endpoint was met, and because patients with a cerebrovascular disease were excluded from study participation, it is unlikely that patients were exposed to excessive health risk.

## Conclusions

O_2_/CO_2_ improves cerebral TOI and PaO_2_ in anesthetized bariatric patients. Better apnea tolerance could not be confirmed.

## Supplementary Information

**Additional file 1:** **Supplemental information S1.** shows the time difference to reach the lowest tissueoxygenation index (TOI = regional oxygen saturation, SrO_2_) value inboth treatment arms. Time of the O_2_/Air treatment has beensubtracted from the corresponding apnea time after O_2_/CO_2_treatment. Positive values indicate a longer apnea time to reach the lowest TOIvalue after O_2_/CO_2_treatment, negative values indicate ashorter apnea time apnea (A). In the subsequent figures, the course ofTOI during apnea after O_2_/Air and after application of O_2_/CO_2_is depicted for all individuals (B). The red lines indicate O_2_/Airtreatment, turquoise lines indicate O_2_/CO_2_ treatment, thefirst intervention is shown in solid, the second intervention in dashed lines.The blue line indicates the lowest TOI value, that was measured in bothtreatment groups.

## Data Availability

De-identified data of the measurements are available from the corresponding author upon reasonable request.
