# Supplementary material for: There is no evidence that carbon dioxide-enriched oxygen before apnea affects the time to arterial desaturation, but it might improve cerebral oxygenation in anesthetized obese patients: a single-blinded randomized crossover trial
Source: BMC Anesthesiol. 2023 Feb 6;23:41. doi: 10.1186/s12871-023-01982-9 (PMC9900199; doi:10.1186/s12871-023-01982-9)
Supplement: Supplementary file 1 — Additional file 1: Supplemental information S1. shows the time difference to reach the lowest tissueoxygenation index (TOI = regional oxygen saturation, SrO2) value inboth treatment arms. Time of the O2/Air treatment has beensubtracted from the corresponding apnea time after O2/CO2treatment. Positive values indicate a longer apnea time to reach the lowest TOIvalue after O2/CO2treatment, negative values indicate ashorter apnea time apnea (A). In the subsequent figures, the course ofTOI during apnea after O2/Air and after application of O2/CO2is depicted for all individuals (B). The red lines indicate O2/Airtreatment, turquoise lines indicate O2/CO2 treatment, thefirst intervention is shown in solid, the second intervention in dashed lines.The blue line indicates the lowest TOI value, that was measured in bothtreatment groups. [file 12871_2023_1982_MOESM1_ESM.docx]

**Supplemental Information S1**

**A**

**
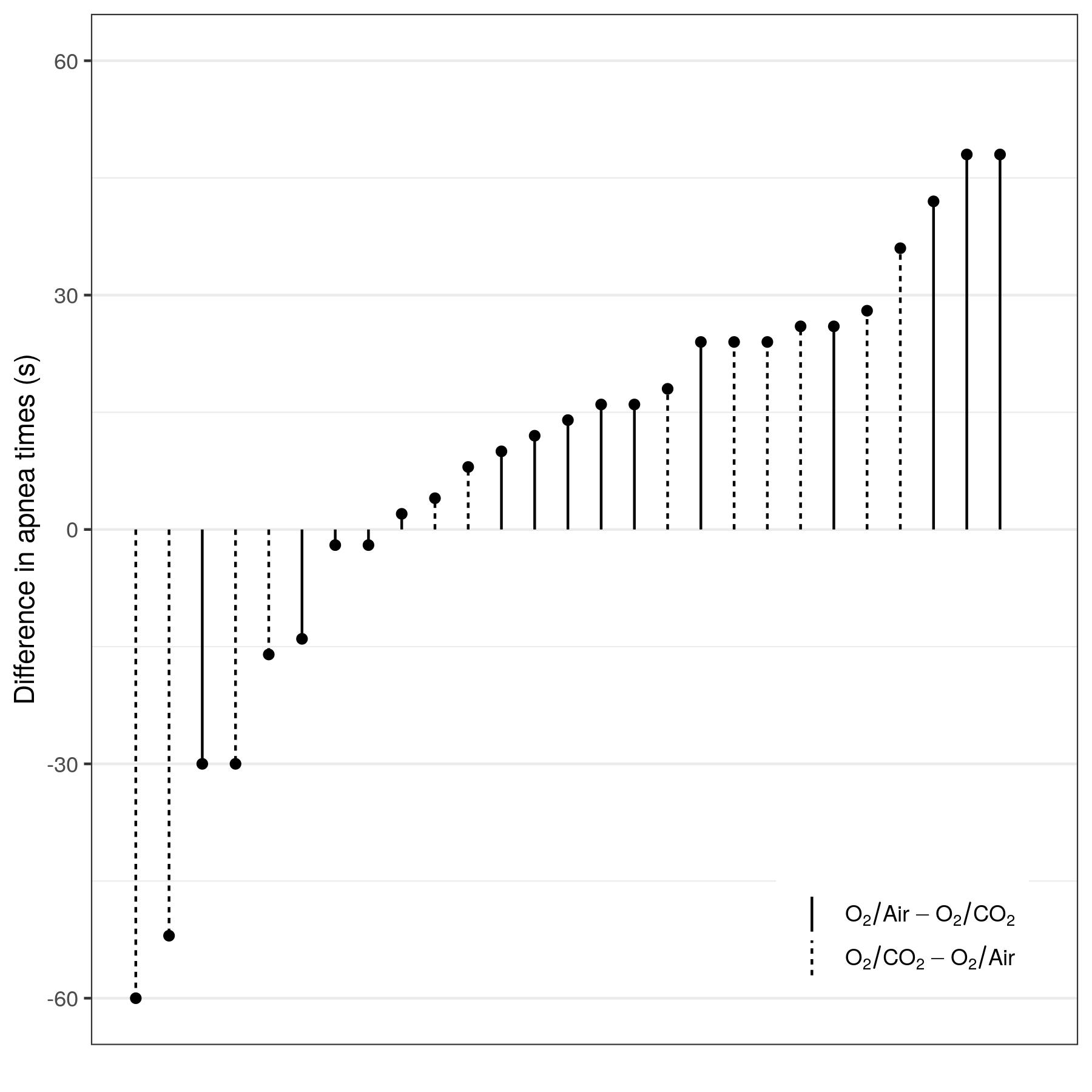
**

**B**

**
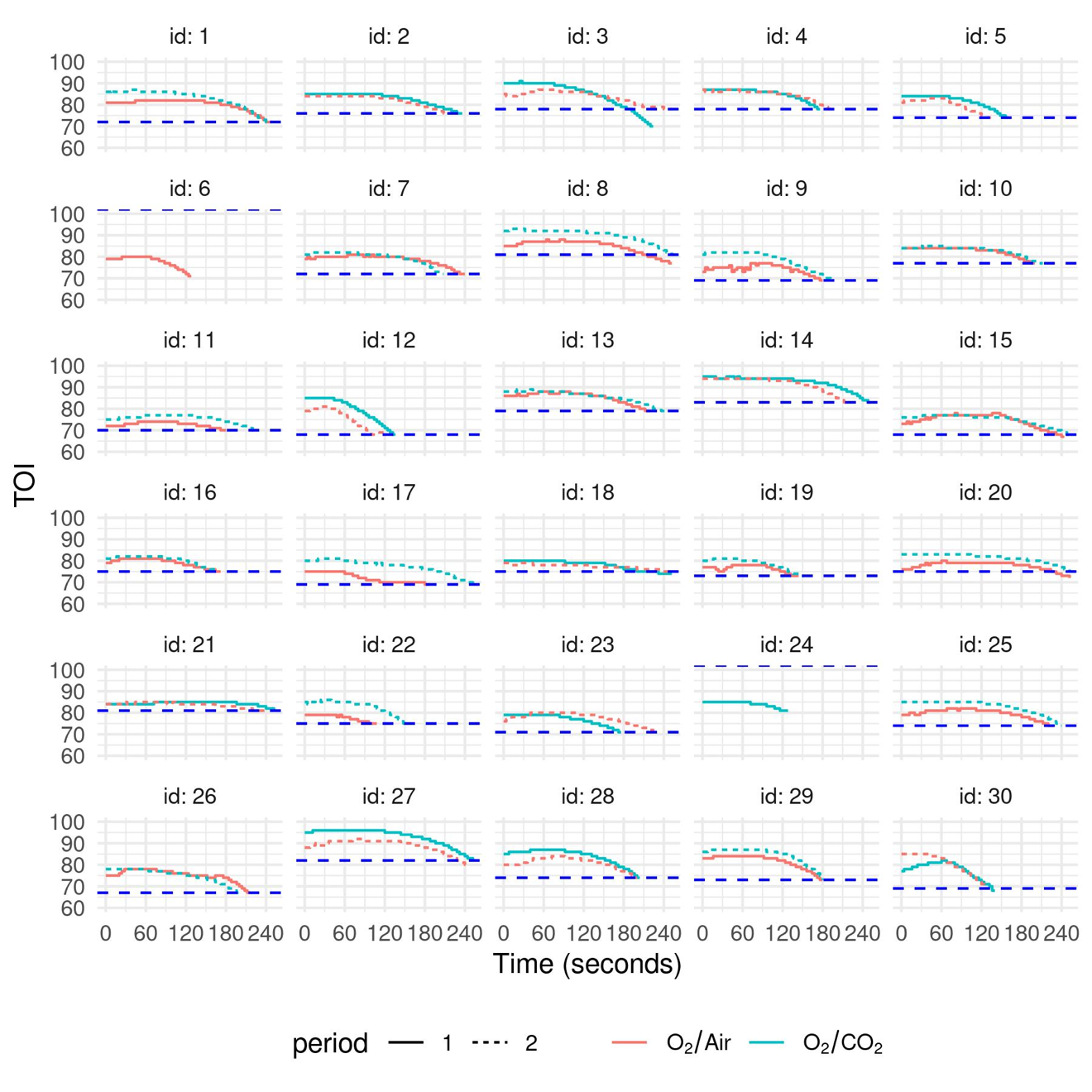
**

**Supplemental information S1** shows the time difference to reach the lowest tissue oxygenation index (TOI = regional oxygen saturation, SrO_2_) value in both treatment arms. Time of the O_2_/Air treatment has been subtracted from the corresponding apnea time after O_2_/CO_2_ treatment. Positive values indicate a longer apnea time to reach the lowest TOI value after O_2_/CO_2_treatment, negative values indicate a shorter apnea time apnea (**A**).

In the subsequent figures, the course of TOI during apnea after O_2_/Air and after application of O_2_/CO_2_ is depicted for all individuals (**B**). The red lines indicate O_2_/Air treatment, turquoise lines indicate O_2_/CO_2_ treatment, the first intervention is shown in solid, the second intervention in dashed lines. The blue line indicates the lowest TOI value, that was measured in both treatment groups.
